# Prevalence and Subtype Analysis of *Blastocystis hominis* Isolated from Patients in the Northeast of Iran

**DOI:** 10.1155/2021/8821885

**Published:** 2021-01-13

**Authors:** Mitra Salehi, Jalal Mardaneh, Hamid Reza Niazkar, Mohammadhaasan Minooeianhaghighi, Elahe Arshad, Fateme Soleimani, Alireza Mohammadzadeh

**Affiliations:** ^1^Department of Parasitology, Faculty of Medicine, Gonabad University of Medical Sciences, Gonabad, Iran; ^2^Student Research Committee, Gonabad University of Medical Sciences, Gonabad, Iran; ^3^Department of Microbiology, Faculty of Medicine, Gonabad University of Medical Sciences, Gonabad, Iran

## Abstract

*Blastocystis hominis* is the most common intestinal parasite found in humans and many other hosts. Pathogenicity of *Blastocystis* spp. remains controversial, and it has been suggested that it may be associated with specific subtypes of the organism. This study identified the *B. hominis* subtypes and their prevalence rates in the northeast of Iran. A total of 1878 samples were collected from the northeast of Iran from January to December 2017. The patients' demographic details were recorded. Samples were examined by a wet mount, and genomic DNA was extracted from positive samples. Also, PCR was done on the positive samples, and sequencing and phylogenetic analysis were subsequently performed. From 1878 collected stool samples, 152 (8.1%) Blastocystis samples were detected by the microscopic method. Of the 152 samples, *Blastocystis* spp. were found in 53.6% of the men and 28.9% of the women who showed clinical gastrointestinal symptoms, and a significant relationship was observed between gender and clinical symptoms (*P* = 0.002). A meaningful relationship was found between the season and infection with this parasite (*P* value = 0.003). The results of the sequencing of 22 PCR products showed the dominance of ST3, which was isolated from 10 (45.45%) patients, while ST1, ST2, and ST7 were found in 4 (18.19%), 7 (31.81%), and 1 (4.55%) patients, respectively. In this study, ST7 had a low prevalence in the northeast of Iran, and similar to previous studies, ST3 was the dominant subtype.

## 1. Introduction

*Blastocystis hominis* is a zoonotic protozoan that affects human and animal intestines and has no specific hosts [1, 2]. *B. hominis* infection may present with various gastrointestinal signs and symptoms including diarrhea, abdominal pain, cramps, and nausea. Moreover, host reaction to *Blastocystis* spp. colonization may lead to eosinophilic enteritis [3, 4]. The prevalence of this parasite has been reported more than 60% in developing countries and between 5% and 20% in developed countries [2, 5]. *Blastocystis* spp. transmit through the oral and fecal routes, and its rate increases with poor hygiene and a high level of animal contact [6]. According to previous studies, this parasite is more common in adults while it becomes more prevalent in some seasons [7, 8]. Given the high prevalence of this parasite in developing countries, more attention has to be paid as it contaminates water and food [9].

Contaminated water is the leading cause of spreading this parasite [10]. Despite extensive studies conducted on the subject, there is little information available about the pathogenesis of this parasite. Some symptoms of infection with *Blastocystis* spp. include nausea, anorexia, bloating, constipation, stomachache, and acute or chronic diarrhea [11–14]. *Blastocystis* species have an extensive genetic variation [15, 16]. In a recent molecular analysis, nine subtypes of *Blastocystis* spp. were isolated from humans, pigs, birds, rodents, and monkeys [17]. The differentiation of *Blastocystis* species is impossible with routine methods such as microscopy, staining, and culture, due to their low sensitivity. The differentiation of these subtypes is performed by Small Subunit Ribosomal RNA Genes (SSUr DNA), and molecular-based methods are necessary for assessing the genetic diversity of these organisms [16]. Molecular epidemiological studies are especially useful for detecting transmission patterns, medication resistance, and specific hosts. The genetic prevalence and diversity of *Blastocystis* have been recently investigated in Iran [18], and molecular PCR-RFLP has been recently used with specific primers for assessing the genetic diversity of *Blastocystis* species [19, 20]. Given the lack of information about the prevalence of *Blastocystis* and its genotypes in the northeast of Iran, the present study investigates the prevalence and genotyping of *Blastocystis* isolated from patients in this region.

## 2. Materials and Methods

In this study, 1878 samples were collected from the northeast of Iran from January to December 2017. The patients' demographic details were recorded. Normal saline and Lugol's iodine smears were prepared from the fecal samples, and ethyl acetate formalin and tri-chrome staining were also carried out [2]. Approximately 200 mg of each positive sample was kept at -20°C until the subsequent molecular analysis.

### 2.1. PCR Assay and Sequencing

DNA was extracted from the fecal samples according to the instructions provided in the GeneAll® Exgene™ Stool DNA mini kit, and the extracted DNA was stored at -20°C. The SSU rDNA gene was proliferated using forward specific primers, Blast 505-532 (5′ GGA GGT AGT GAC AAT AAATC 3′; forward) and Blast 998-1017 (5′ TGC TTT CGC ACT TGT TCATC 3′; reverse) [21]. These primers approximately amplified a 500 bp fragment. PCR reaction was performed in 30 *μ*l Ampliqone (Taq DNA Polymerase Master Mix RED, Denmark). Twenty-five microliters of the Taq Master mix were used with ten ng template DNA, 0.1 *μ*M of each primer, and distilled water. Cycles of PCR were set up as the following: predenaturation step at 94°C for 3 min and 33 cycles of denaturation at 95°C for 35 s, annealing at 56°C for 45 s and extension at 72°C for 1 min with an elongation step of 5 min at 72°C at the last cycle. A total of 22 samples were selected randomly from different age groups (Table 1) and sent to the Bioneer Korea Company to determine the nucleotide sequence.

### 2.2. Analysis Sequencing

The analysis of DNA sequencing was carried out on 22 positive PCR samples. The SSU rDNA sequences obtained were compared with the gene sequences available at the gene bank using the BLAST program (http://www.ncbi.nlm.nih.gov/BLAST/): ST-1|HQ641595, ST-2|HQ641605, ST-3|HQ641611, ST-4|HQ641621, ST-5|HQ641630, ST-6|HQ641658, ST-7|HQ641661, ST-5|HQ641630, ST-6|HQ641658, and ST-7|HQ641661. The distance-based analysis was conducted on the sequencing data by using molecular evolutionary genetic analysis version 4 (MEGA 4) [22], and a phylogenetic tree was constructed using the neighbor-joining method [23] with the Kimura 2-parameter model [24]. The support of monophyletic groups was assessed by the bootstrap method with 1,000 replicates [25].

### 2.3. Ethical Considerations

The current study was approved by the Research Council and the Ethics Committee of the Gonabad University of Medical Sciences, and written informed consent was also obtained from participants before the study.

### 2.4. Statistical Analysis

Frequency distributions of strains and demographic information were analyzed by descriptive statistical methods, and qualitative variables were analyzed by the chi-square test.

## 3. Results

**Figure 1:**
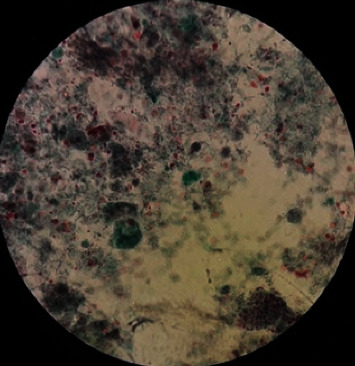
The presence of *Blastocystis* spp. in the background with tri-chrome staining.

**Figure 2:**
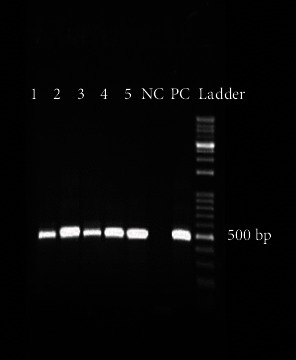
PCR-product of *Blastocystis* isolates. Lanes 1 to 5: *Blastocystis* isolates; lane NC: negative control; lane PC: positive control; DNA ladder, 100 bp.

**Figure 3:**
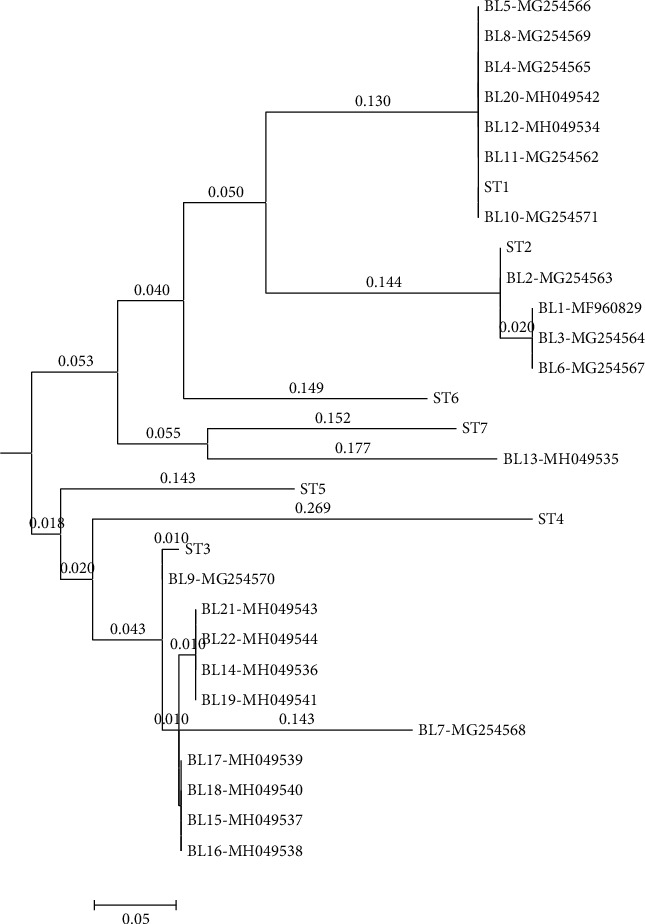
Phylogenetic tree of the SSU-rDNA gene sequences of *Blastocystis* specimens. The phylogenetic tree was inferred using the neighbor-joining method.

**Figure 4:**
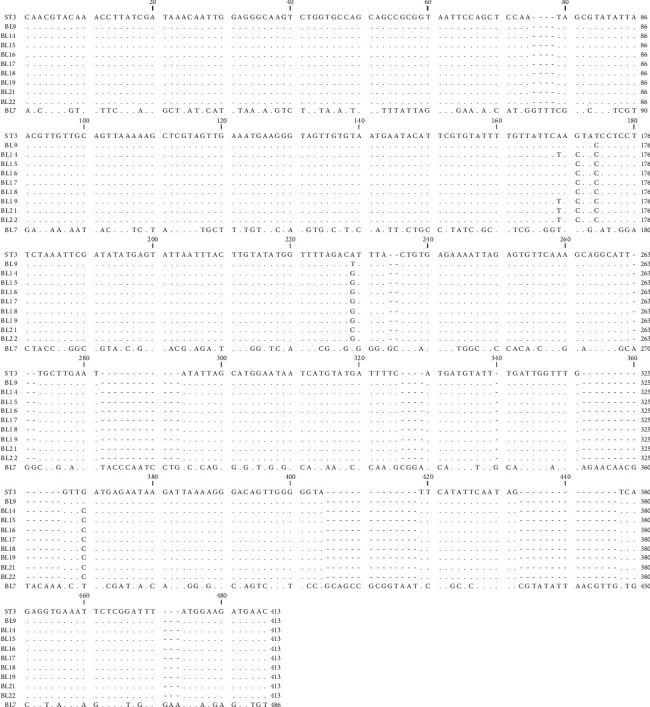
Alignment nucleotides of accession number MG254568 (BL7) with the ST3 reference.

**Figure 5:**
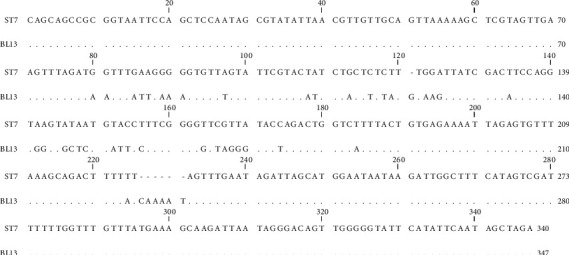
Alignment nucleotides of accession number MH049535 (BL13) with the ST7 reference.

From 1878 stool samples, *Blastocystis* spp. were detected in 152 (8.1%) ones by the microscopic method (Figure 1). The samples were collected from 991 (76.5%) women and 887 (47.2%) men, and the prevalence of this parasite was 7.7% in women and 37.8% in men, with no significant relationship between gender and *Blastocystis* infection (Chi = 0.22, *P* = 0.63) (Table 1). Most positive cases were found in the age group of 30-39 years old, and a significant relationship was observed between the age groups and infection with this parasite (Chi = 13.90, *P* = 0.01) (Table 2). Of the 152 samples, *Blastocystis* spp. were found in 53.6% of the men and 28.9% of the women who showed clinical gastrointestinal symptoms, and a significant relationship was observed between gender and clinical symptoms (Chi = 9.57, *P* = 0.002) (Table 3). This parasite was more prevalent in the summer and spring (Table 4), and a significant relationship was found between the season and infection with this parasite (Chi = 13.89, *P* value = 0.003). In the present study, other parasitic factors were also isolated in addition to *Blastocystis* spp. (Table 5). All the positive cases obtained by the microscopic method were also confirmed by the molecular method (Figure 2). The results of the sequencing of 22 PCR products (Table 2) showed the dominance of ST3, which was isolated from 10 (45.45%) patients, while ST1, ST2, and ST7 were found in 4 (18.19%), 7 (31.81%), and 1 (4.55%) patients, respectively. The nucleotide sequences of the 22 sequenced and subtyped samples from the present study have been deposited in GenBank under accession numbers MG254562-MG254571, MH049534-MH049544, and MF960829. The phylogenic tree of the isolates is presented in Figure 3. Given the alignment, nucleotides deposited as MG254568 (BL7) were considerably different from the rest of the accession numbers in this branch (Figure 4). Also, the accession number MH049535 (BL13) was located in one branch with the ST7 reference.  Figure 5 presents its nucleotide difference.

## 4. Discussion

The prevalence of *Blastocystis* spp. found by the microscopic method was 8.09%, which concurs with the previous studies conducted in Iran [18, 26–28]. The prevalence of *Blastocystis* spp. has been reported as 60% in developing countries and 5% to 20% in developed countries [2, 5, 18]. According to previous studies, the prevalence of *Blastocystis* spp. is 8.1% in semidesert regions [28]. The present study was conducted in the northeast of Iran, which is a semidesert region, and the prevalence of this parasite was found 8.1% in this region, which agrees with the prevalence found in regions with insufficient humidity and poor temperatures [29–31].

In the present study, the majority of the positive cases were observed in the age group of 30-39 years old, while the studies conducted in Turkey and Libya showed a significant relationship between age group and infection [32, 33], and 8.37% of men were infected with *Blastocystis* spp. Other studies have also reported higher infection rates in men compared to women [18, 32–34], but no significant relationship was found between gender and infection with this parasite (*P* = 0.63). Khalili et al. did not find a significant relationship between gender and infection [35]. Likewise, no significant relationship was observed between gender and age and infection with *Blastocystis* spp. or between clinical symptoms and infection with this parasite in Australia [36]. A notable case examined in the present study was clinical symptoms in men and women. The prevalence of clinical symptoms was higher in men (53.6%) than in women (28.9%), and a significant relationship was found between gender and clinical symptoms (*P* = 0.002). Like in many other studies, in the present study, the prevalence of *Blastocystis* spp. was higher in the summer and spring compared to other seasons, and a significant relationship (*P* value = 0.003) was also observed between the prevalence of this parasite and the season [18, 37–39]. In the present study, the prevalence of other detected parasites was as follows: *Entamoeba coli* (6.70%), *Giardia* (5.05%), *Iodamoeba bütschlii* (4.47%), and *Entamoeba histolytica* (0.15%) (Table 5).

The sequencing results showed that ST3 (45.45%) was the dominant subtype, while ST7 was isolated from only one patient. In Iran, only Khoshnood et al. (2015) have reported the presence of the ST7 subtype, which indicates its low prevalence in Iran compared to the other subtypes. This subtype has been isolated in a few studies in the Middle East [18, 40]. Previous studies conducted in Iran have isolated ST1, ST2, ST3, ST4, ST5, ST6, and ST7 from humans [28]. In the present study, ST1, ST2, ST3, and ST7 were isolated from the patients, while no cases of ST4, ST5, ST6, ST8, and ST9 were reported. Like in many studies, ST3 was more prevalent than the other subtypes in the present study [8, 18, 41, 42]. Studies conducted in Iran have shown that ST1 and ST2 were the most prevalent subtypes after ST3 [16, 19, 20, 28, 43]. Similarly, ST1 was the dominant subtype after ST3 in this study. The prevalence of ST1 and ST3 is also high in neighboring countries of Iran and also in countries outside the Middle East [44–48].

The present study revealed a sample with the accession number MG254568 in the phylogenic tree in subtype 3, but as shown in the phylogenic tree, this sample is located away from other samples in the branch, and the nucleotide alignment of this sample is considerably different from the other numbers registered under the ST3 reference. Moreover, given the phylogenic tree and the nucleotide alignment, a sample with the accession number MH049535 was located in the ST7 group.

## 5. Conclusions

According to the present findings, gender should be taken into account in assessing infection with *Blastocystis* and its clinical symptoms. Also, ST7 has a low prevalence in different parts of Iran, and the subtype was also isolated in the present study, and ST3 was the dominant subtype.

## Figures and Tables

**Table 1:** The total number of men and women infected with *Blatocystis* spp.

Sex	Infection		Total
	Positive	Negative	
Men	69 (7.8%)	818 (92.2%)	887 (47.2%)
Women	83 (8.4%)	908 (91.6%)	991 (52.8%)
Total	152 (8.1%)	1726 (91.9%)	1878 (100%)

**Table 2:** The rate of *Blastocystis* spp. infection in different age groups and the number of sequenced cases in each group.

Age groups	Infection		Total	Total number of sequences
	Positive	Negative		
<20	30 (9.5%)	336 (90.5%)	366 (20.0%)	5
20-29	32 (9.1%)	344 (90.9%)	376 (22.2%)	4
30-39	45 (12.5%)	414 (87.5%)	459 (22.8%)	7
40-49	18 (5.2%)	375 (94.8%)	393 (21.7%)	2
50-59	13 (12.1%)	144 (87.9%)	157 (6.8%)	2
>60	14 (13.7%)	113 (86.3%)	127 (6.5%)	2
Total	152 (9.6%)	1726 (90.4%)	1878 (100%)	22

**Table 3:** The percentage of clinical symptoms in men and women.

Sex	Symptom		Total
	Positive	Negative	
Men	37 (53.6%)	32 (46.4%)	69 (45.4%)
Women	24 (28.9%)	59 (71.1%)	83 (54.6%)
Total	61 (40.1%)	91 (59.9%)	152 (100%)

**Table 4:** The rate of *Blastocystis* spp. infection in different season.

Season	Infection		Total
	Positive	Negative	
Spring	54 (8.7%)	570 (91.3%)	624 (33.2%)
Summer	67 (10.6%)	564 (89.4%)	631 (33.6%)
Fall	17 (5.3%)	305 (94.7%)	322 (17.1%)
Winter	14 (4.7%)	287 (95.3%)	301 (16.0%)
Total	152 (8.1%)	1726 (91.9%)	1878 (100%)

**Table 5:** The percentage of other parasites in the patients.

Parasite type	*Entamoeba coli*	*Giardia*	*Iodamoeba bütschlii*	*Entamoeba histolytica*	*Blastocystis* spp.
Frequency and percentage	126 (6.70)	95 (5.05)	84 (4.47)	3 (0.15)	152 (8.09)

## Data Availability

The data used in this study are included within the article.

## References

[1] Tan K. S. W. (2004). *Blastocystis* in humans and animals: new insights using modern methodologies. *Veterinary Parasitology*.

[2] Stenzel D. J., Boreham P. F. (1996). *Blastocystis* hominis revisited. *Clinical Microbiology Reviews*.

[3] Bilinski J., Lis K., Tomaszewska A. (2020). Eosinophilic gastroenteritis and graft-versus-host disease induced by transmission of Norovirus with fecal microbiota transplant. *Transplant Infectious Disease*.

[4] Ahmadabadi F. B., Ghorbani M., Niazkar H. R. (2019). Eosinophilic gastroenteritis and colitis with elevated level of serum IgA: a case report. *Govaresh*.

[5] Pegelow K., Gross R., Pietrzik K., Lukito W., Richards A. L., Fryauff D. J. (1997). Parasitological and nutritional situation of school children in the Sukaraja district, West Java, Indonesia. *The Southeast Asian Journal of Tropical Medicine and Public Health*.

[6] Ustün S., Turgay N. (2006). Blastocystis hominis and bowel diseases. *Turkish Journal of Parasitology*.

[7] Su F.-H., Chu F.-Y., Li C.-Y. (2009). Blastocystis hominis infection in long-term care facilities in Taiwan: prevalence and associated clinical factors. *Parasitology Research*.

[8] Hussein E. M., Hussein A. M., Eida M. M., Atwa M. M. (2008). Pathophysiological variability of different genotypes of human *Blastocystis hominis* Egyptian isolates in experimentally infected rats. *Parasitology Research*.

[9] Tan K. S. W. (2008). New insights on classification, identification, and clinical relevance of *Blastocystis* spp. *Clinical Microbiology Reviews*.

[10] Kuo H.-Y., Chiang D.-H., Wang C.-C. (2008). Clinical significance of *Blastocystis hominis*: experience from a medical center in northern Taiwan. *Journal of Microbiology, Immunology, and Infection*.

[11] El-Shazly A. M., Abdel-Magied A. A., El-Beshbishi S. N., El-Nahas H. A., Fouad M. A. H., Monib M. S. M. (2005). Blastocystis hominis among symptomatic and asymptomatic individuals in Talkha Center, Dakahlia Governorate, Egypt. *Journal of the Egyptian Society of Parasitology*.

[12] Kaya S. C. E., Aridogan B. C., Arikan S., Demirci M. (2007). Pathogenicity of *Blastocystis hominis,* a clinical reevaluation. *Türkiye Parazitolojii Dergisi*.

[13] Suresh K., Smith H. (2004). Comparison of methods for detecting *Blastocystis hominis*. *European Journal of Clinical Microbiology & Infectious Diseases*.

[14] Tasova Y., Sahin B., Koltas S., Paydas S. (2000). Clinical significance and frequency of *Blastocystis hominis* in Turkish patients with hematological malignancy. *Acta Medica Okayama*.

[15] Clark C. G. (1997). Extensive genetic diversity in *Blastocystis hominis*. *Molecular and Biochemical Parasitology*.

[16] Sardarian K., Hajilooi M., Maghsood A., Moghimbeigi A., Alikhani M. (2012). A study of the genetic variability of *Blastocystis hominis* isolates in Hamadan, west of Iran. *Jundishapur Journal of Microbiology*.

[17] Stensvold C. R., Suresh G. K., Tan K. S. W. (2007). Terminology for *Blastocystis* subtypes - a consensus. *Trends in Parasitology*.

[18] Khoshnood S., Rafiei A., Saki J., Alizadeh K. (2015). Prevalence and genotype characterization of *Blastocystis hominis* among the Baghmalek people in southwestern Iran in 2013 - 2014. *Jundishapur Journal of Microbiology*.

[19] Moosavi A., Haghighi A., Mojarad E. N. (2012). Genetic variability of *Blastocystis* sp. isolated from symptomatic and asymptomatic individuals in Iran. *Parasitology Research*.

[20] Motazedian H., Ghasemi H., Sadjjadi S. M. (2013). Genomic diversity of *Blastocystis hominis* from patients in southern Iran. *Annals of Tropical Medicine and Parasitology*.

[21] Bohm-Gloning B., Knobloch J., Walderich B. (1997). Five subgroups of *Blastocystis hominis* from symptomatic and asymptomatic patients revealed by restriction site analysis of PCR-amplified 16S-like rDNA. *Tropical Medicine & International Health*.

[22] Tamura K., Dudley J., Nei M., Kumar S. (2007). MEGA4: molecular evolutionary genetics analysis (MEGA) software version 4.0. *Molecular Biology and Evolution*.

[23] Saitou N., Nei M. (1987). The neighbor-joining method: a new method for reconstructing phylogenetic trees. *Molecular Biology and Evolution*.

[24] Kimura M. (1980). A simple method for estimating evolutionary rates of base substitutions through comparative studies of nucleotide sequences. *Journal of Molecular Evolution*.

[25] Felsenstein J. (1985). Phylogenies and the comparative method. *The American Naturalist*.

[26] Daryani N. B. G., Ettehad M., Sharif M. H., Dehghan A. (2006). A cross-sectional study of *Blastocystis hominis* in primary school children, Northwest Iran. *International Journal of Tropical Medicine*.

[27] Haghighi A., Khorashad A. S., Mojarad E. N., Kazemi B., Nejad M. R., Rasti S. (2009). Frequency of enteric protozoan parasites among patients with gastrointestinal complaints in medical centers of Zahedan, Iran. *Transactions of the Royal Society of Tropical Medicine and Hygiene*.

[28] Javanmard E., Niyyati M., Ghasemi E., Mirjalali H., Asadzadeh Aghdaei H., Zali M. R. (2018). Impacts of human development index and climate conditions on prevalence of *Blastocystis*: a systematic review and meta-analysis. *Acta Tropica*.

[29] Duda A., Kołodziejczyk L., Lanocha A., Kosik-Bogacka D., Lanocha-Arendarczyk N. (2015). The prevalence of *Blastocystis hominis* and other protozoan parasites in soldiers returning from peacekeeping missions. *The American Journal of Tropical Medicine and Hygiene*.

[30] Pandey P. K., Verma P., Marathe N. (2015). Prevalence and subtype analysis of *Blastocystis* in healthy Indian individuals. *Infection, Genetics and Evolution*.

[31] Cabrine-Santos M., do Nascimento Cintra E., do Carmo R. A. (2015). Occurrence of Blastocystis spp. in Uberaba, Minas Gerais, Brazil. *Revista do Instituto de Medicina Tropical de São Paulo*.

[32] Beyhan Y., Yilmaz H., Cengiz Z., Ekici A. (2015). Clinical significance and prevalence of Blastocystis hominis in Van, Turkey. *Saudi Medical Journal*.

[33] Abdulsalam A. M., Ithoi I., Al-Mekhlafi H. M. (2013). Prevalence, predictors and clinical significance of Blastocystis sp. in Sebha, Libya. *Parasites & Vectors*.

[34] Badparva E., Pournia Y., Fallahi S. H. (2012). Prevalence of *Blastocystis hominis* in Lorestan Province, West of Iran, 2010. *Asian Journal of Biological Sciences*.

[35] Khalili B. K. M. (2012). Taghipour s. *Blastocystis Hominis* infection among hospitalized children due to diarrhea in Hajar Hospital, Shahre-Kord, Iran. *Archives of Clinical Infectious Diseases*.

[36] Leder K., Hellard M. E., Sinclair M. I., Fairley C. K., Wolfe R. (2005). No correlation between clinical symptoms and *Blastocystis hominis* in immunocompetent individuals. *Journal of Gastroenterology and Hepatology*.

[37] Al-Fellani M. A., Khan A. H., Al-Gazoui R. M., Zaid M. K., Al-Ferjani M. A. (2007). Prevalence and clinical features of *Blastocystis hominis* infection among patients in Sebha, Libya. *Sultan Qaboos University Medical Journal*.

[38] Salehi R., Haghighi A., Stensvold C. R. (2017). Prevalence and subtype identification of *Blastocystis* isolated from humans in Ahvaz, Southwestern Iran. *Gastroenterology and Hepatology from Bed to Bench*.

[39] El Safadi D., Cian A., Nourrisson C. (2016). Prevalence, risk factors for infection and subtype distribution of the intestinal parasite *Blastocystis sp.* from a large-scale multi-center study in France. *BMC Infectious Diseases*.

[40] Yakoob J., Jafri W., Beg M. A. (2010). Irritable bowel syndrome: is it associated with genotypes of *Blastocystis hominis*. *Parasitology Research*.

[41] Tan T. C., Ong S. C., Suresh K. G. (2009). Genetic variability of *Blastocystis* sp. isolates obtained from cancer and HIV/AIDS patients. *Parasitology Research*.

[42] Iseki M., Ali I. K. M. D., Hossain M. B. (2004). Polymerase chain reaction-based genotype classification among human *Blastocystis hominis* populations isolated from different countries. *Parasitology Research*.

[43] Khademvatan S., Masjedizadeh R., Yousefi-Razin E. (2018). PCR-based molecular characterization of *Blastocystis hominis* subtypes in southwest of Iran. *Journal of Infection and Public Health*.

[44] Abu-Madi M., Aly M., Behnke J. M., Clark C. G., Balkhy H. (2015). The distribution of *Blastocystis* subtypes in isolates from Qatar. *Parasites & Vectors*.

[45] Dagci H., Kurt Ö., Demirel M. (2014). Epidemiological and diagnostic features of *Blastocystis* infection in symptomatic patients in Izmir province, Turkey. *Iranian Journal of Parasitology*.

[46] Dogruman-Al F., Simsek Z., Boorom K. (2010). Comparison of methods for detection of *Blastocystis* infection in routinely submitted stool samples, and also in IBS/IBD patients in Ankara, Turkey. *PLoS One*.

[47] Khan Z. A., Alkhalife I. S. (2005). Prevalence of *Blastocystis hominis* among "healthy" food handlers in Dammam, Saudi Arabia. *Journal of the Egyptian Society of Parasitology*.

[48] Souppart L., Moussa H., Cian A. (2010). Subtype analysis of *Blastocystis* isolates from symptomatic patients in Egypt. *Parasitology Research*.

